# Selective Residual Action Chunking for Vision-Guided Multi-Task Robotic Manipulation

**DOI:** 10.3390/s26216992

**Published:** 2026-11-05

**Authors:** Yibo Cao, Jiahong Huang, Zijun Yin

**Affiliations:** School of Artificial Intelligence, South China Normal University, Foshan 528225, China; 2025024779@m.scnu.edu.cn (J.H.); 2025024758@m.scnu.edu.cn (Z.Y.)

**Keywords:** visual sensing, vision-based robot control, multi-task robotic manipulation, imitation learning, action chunking, residual learning

## Abstract

Vision-guided robotic manipulation requires control actions that reflect visual observations, robot state, and task goals. Action Chunking with Transformers (ACT) performs well on individual tasks, but shared multi-task policies must accommodate different action distributions and state-dependent control requirements. We propose an ACT-based selective residual action-chunking policy using global and wrist RGB images together with proprioceptive feedback. A task-conditioned ACT model is first trained on multi-task demonstrations. Its parameters are then frozen while bounded residual experts learn candidate action corrections. A gain predictor uses observation-conditioned decoder features, robot state, task information, and base-action statistics to select a correction or retain the base prediction. Gain supervision is derived from reductions in imitation error. The robot executes an action prefix and updates its prediction from new observations, incorporating selective correction into closed-loop manipulation. Across all ten LIBERO-Goal tasks, the method achieves a mean success rate of 91.2%, exceeding MTACT and BAKU by 11.4 and 4.2 percentage points. On three jointly learned real-world tasks, it achieves 78.7%, exceeding MTACT by 10.0 percentage points. These results support selective residual action generation for vision-guided multi-task robot control.

## 1. Introduction

Robotic manipulation in manufacturing, logistics, and domestic assistance depends on converting observations of the workspace and robot into appropriate control actions. Visual sensing provides information about object appearance, spatial relationships, and the progress of an operation, while proprioceptive feedback describes the robot’s configuration. The connection between these measurements and action generation is central to grasping and manipulation [1,2]. When one robot performs several tasks in a shared workspace, its controller must interpret the observations in relation to the intended goal and update its actions as the interaction evolves.

Visual feedback has long supported robot control through image-based and position-based visual servoing [3]. Recent studies also examine image feature prediction for discrete-time control [4] and learned mappings from visual representations to control commands [5]. Alongside these developments, visuomotor imitation learning uses demonstrations to learn the relationship between observations and actions [6]. This approach is useful for manipulation sequences involving several objects or successive operations, for which specifying all intermediate visual targets and control rules can be laborious. Research on sensor modalities and fusion in learned robot policies further emphasizes that visual and proprioceptive inputs must be considered as part of the action-generation loop [7].

Action Chunking with Transformers (ACT) is a representative visuomotor imitation-learning method. It combines a Transformer with a conditional variational autoencoder to predict continuous action chunks, exploiting temporal relationships between actions and modeling variation in demonstrations. Its performance on individual fine-grained manipulation tasks demonstrates the value of action chunking [8]. In a multi-task setting, however, similar observations can correspond to different target objects, destinations, or operation sequences. Shared parameters must accommodate these different action distributions, creating the potential for task interference and negative transfer [9]. Task information helps distinguish goals, but the controller must still generate suitable actions for the current visually observed scene and robot configuration.

Existing methods address this problem through task conditioning and policy structure. RoboAgent introduces task-language conditioning into its multi-task action-chunking Transformer (MTACT) [10], while BAKU studies observation encoding, information fusion, and action prediction in a unified multi-task policy [11]. Multi-view and temporal representations provide additional ways to organize observations for manipulation [12]. Conditional architectures offer another direction: MoE-ACT introduces sparse task-conditioned experts into ACT [9], and Skill Mixture-of-Experts Policy (SMP) composes actions from reusable skill representations [13]. These studies motivate adapting action generation to task and observation context. We investigate a complementary question: how can a trained multi-task visuomotor policy be refined through conditional corrections to its existing action predictions?

We address this question using a selective residual action-chunking policy. Global and wrist RGB images, proprioceptive state, and task information condition a shared ACT model. After this base model has been trained, its parameters remain fixed while bounded residual experts learn alternative corrections. A gain predictor uses the base decoder features, proprioceptive state, task representation, and base-action statistics to estimate the benefit of each expert relative to the base prediction. The supervision is the reduction in demonstration imitation error. At inference, a residual is applied only when the highest predicted gain exceeds a threshold; otherwise, the base action is retained. The robot executes part of the selected action chunk and then incorporates new observations into the next prediction.

This design places conditional correction within the visual feedback loop. Image information reaches the residual module through the base decoder representation, while proprioception supplies the robot configuration for correction and selection. Keeping the base model fixed concentrates the second training stage on action refinement. Chunk-level selection also maintains a consistent correction branch during the executed action segment. The method thus studies how sensed observations can guide selective action refinement in a learned multi-task controller.

The main contributions are as follows:

1. We propose an ACT-based selective residual policy for vision-guided multi-task manipulation, combining a shared policy conditioned on dual-view RGB observations and proprioceptive feedback with bounded action corrections.
2. We introduce gain supervision relative to the base prediction and observation-conditioned chunk-level selection, integrating expert selection and base-action retention into receding-horizon execution.
3. We evaluate the policy on all ten LIBERO-Goal tasks [14] and three real-world tasks, achieving mean success rates of 91.2% and 78.7%. Comparisons, ablations, and execution analysis examine its effectiveness under the evaluated visual sensing and control configurations.

## 2. Related Work

### 2.1. Visual Sensing and Feedback Control for Manipulation

Visual sensing connects scene information to the control decisions required for robotic grasping and manipulation. Wang et al. reviewed how object features support grasp generation under uncertain information [1]. Cherubini and Navarro-Alarcon organized sensor-based robot control around the measurements, control objectives, and integration of different sensing modalities [2]. Classical visual servoing uses image features or estimated poses to regulate robot motion [3]. Liu et al. combined adaptive image feature prediction with manipulator dynamics in discrete-time visual servoing, addressing the temporal relationship between visual updates and control [4]. These approaches illustrate the importance of incorporating observations into the execution loop, although explicit visual features and geometric control objectives do not directly specify a diverse set of learned manipulation sequences.

Learning-based methods extend the observation-to-control mapping. Botezatu et al. investigated early fusion of RGB images and derived feature maps for predicting visual-servo control commands [5]. Lu et al. combined historical observations, actions, and multi-view attention for robotic manipulation [12]. Ko reviewed sensor modalities and fusion in vision-language-action models, including the relationship between sensor representations and deployment in a control loop [7]. These studies motivate considering scene observations, robot state, and action generation jointly. Our policy uses global and wrist RGB views with proprioceptive state, and refines the resulting action predictions through conditional residuals.

Observation encoding and action generation can also be adapted separately. In simDP, Jung et al. retained a simulation-trained action decoder and adapted the real-world observation encoder within a shared action representation [15]. Our setting instead uses demonstrations from each evaluation domain to train its own multi-task policy. We hold the trained base policy fixed and learn corrections at its action output, focusing on multi-task control under the available observations.

### 2.2. Multi-Task Visuomotor Action-Sequence Learning

Visuomotor imitation learning learns control from paired observations and demonstrated actions [6]. ACT predicts future action chunks using a Transformer and a conditional variational autoencoder [8]. Diffusion Policy generates action sequences through conditional denoising and uses receding-horizon execution to incorporate updated visual observations [16]. Both approaches use temporal action structure while allowing predictions to be refreshed during execution. This structure provides a basis for multi-step manipulation, although sharing an action model across tasks introduces additional demands on task conditioning and action prediction.

MTACT, introduced in RoboAgent, incorporates task-language conditioning into ACT, allowing a shared policy to generate actions for different task goals. RoboAgent also uses semantic augmentation to improve generalization [10]. BAKU studies observation encoding, information fusion, and action prediction for Transformer-based multi-task policies [11]. Sun et al. used language-guided routing to choose synchronous or asynchronous dual-arm policy branches, separating different coordination modes [17]. These methods show how task information can organize the mapping from observations to actions. Our method retains a task-conditioned ACT as the shared base and learns state-dependent residuals, allowing action refinement to be trained separately from the base visuomotor mapping.

### 2.3. Conditional Experts and Residual Policy Learning

Mixture-of-experts models use input-dependent routing to allocate computation among sub-networks [18,19]. In robotic manipulation, MoE-ACT places sparse experts inside the ACT encoder and combines task-conditioned modulation with multi-scale visual information [9]. SMP learns reusable skill representations with a diffusion policy and employs persistent routing to promote temporal consistency [13]. These designs motivate conditional processing of task and observation context. We place experts in the action-residual space and keep the selected expert fixed within each action chunk, while retaining the base prediction as an available output.

Residual policy learning combines an existing controller with learned action corrections. Johannink et al. combined feedback control with residual reinforcement learning for robot manipulation [20]. Silver et al. study residual learning on top of hand-designed policies and model predictive controllers [21]. Staessens et al. investigated constrained residual reinforcement learning for cascaded mechatronic control, using bounded corrections to regulate the interaction between the base controller and learned compensation [22]. These studies provide the motivation for preserving an existing controller while learning to refine its output.

Our approach learns residual action chunks from demonstrations. Demonstrated actions provide both targets for the experts and a common reference for comparing corrected and base predictions. A gain predictor then links these comparisons to the current observation-conditioned representation, task, and robot state. This combines expert selection with base-action retention, extending residual learning to selective action-chunk refinement in a multi-task visuomotor policy.

## 3. Method

We decompose the multi-task policy into shared base action generation and conditional residual correction. A task-conditioned ACT model learns base actions from demonstrations pooled across tasks. Residual experts learn candidate corrections with the base policy fixed, and a gain predictor controls their selection and application according to the task and current state. We describe the action-chunk formulation, residual candidates, gain-guided selection, and two-stage training below.

### 3.1. Sensing Inputs and Closed-Loop Framework

Consider demonstrations for *M* tasks, denoted by $D={\left\{D_{m}\right\}}_{m=1}^{M}$, where $D_{m}$ contains the trajectories for task *m*. A training sample consists of an observation and its subsequent action window. At decision time *t*, the robot receives dual-view RGB images $I_{t}$, proprioceptive state $q_{t}\in R^{d_{q}}$, and a task-language representation $e_{m}\in R^{d_{e}}$. Conditioned on these inputs, the policy predicts an action chunk of length *H*:

(1) $${\hat{A}}_{t}=\pi \left(I_{t},q_{t},e_{m}\right)\in R^{H\times d_{a}}.$$

Here, $d_{q}$, $d_{e}$, and $d_{a}$ denote the dimensions of the state, task representation, and action, respectively. The robot executes the first *h* actions, with 1≤h≤H, before predicting a new chunk from updated observations. Action and state normalization statistics are computed from the training trajectories only. All action errors and residuals below are defined in the normalized action space.

This execution procedure follows a receding-horizon scheme: the policy predicts *H* future actions from the current observation, executes only the first *h*, and generates a new chunk from updated observations, replacing the unexecuted portion of the previous prediction. The base actions, gain estimates, and branch selection are recomputed at each update. One branch is used within a chunk, while successive chunks may use different branches. Here, *H* counts action steps, and its physical duration depends on the control period. In our simulation configuration, the control frequency is 20 Hz, corresponding to 0.05 s per step. A 75-step prediction spans approximately 3.75 s, whereas the eight executed steps span 0.4 s, after which a new action chunk is predicted from updated observations.

The two RGB views observe the workspace and the region near the gripper, respectively, while proprioceptive feedback specifies the robot configuration. These inputs describe the execution state; the task representation specifies the intended operation. Visual information is encoded by the base policy and contributes to its decoder features. The residual module receives these features together with explicit state and task inputs, linking candidate corrections and selection to the current observation. Each new decision follows the same observation-to-action path after the preceding action prefix has been executed.

**Figure 1:**
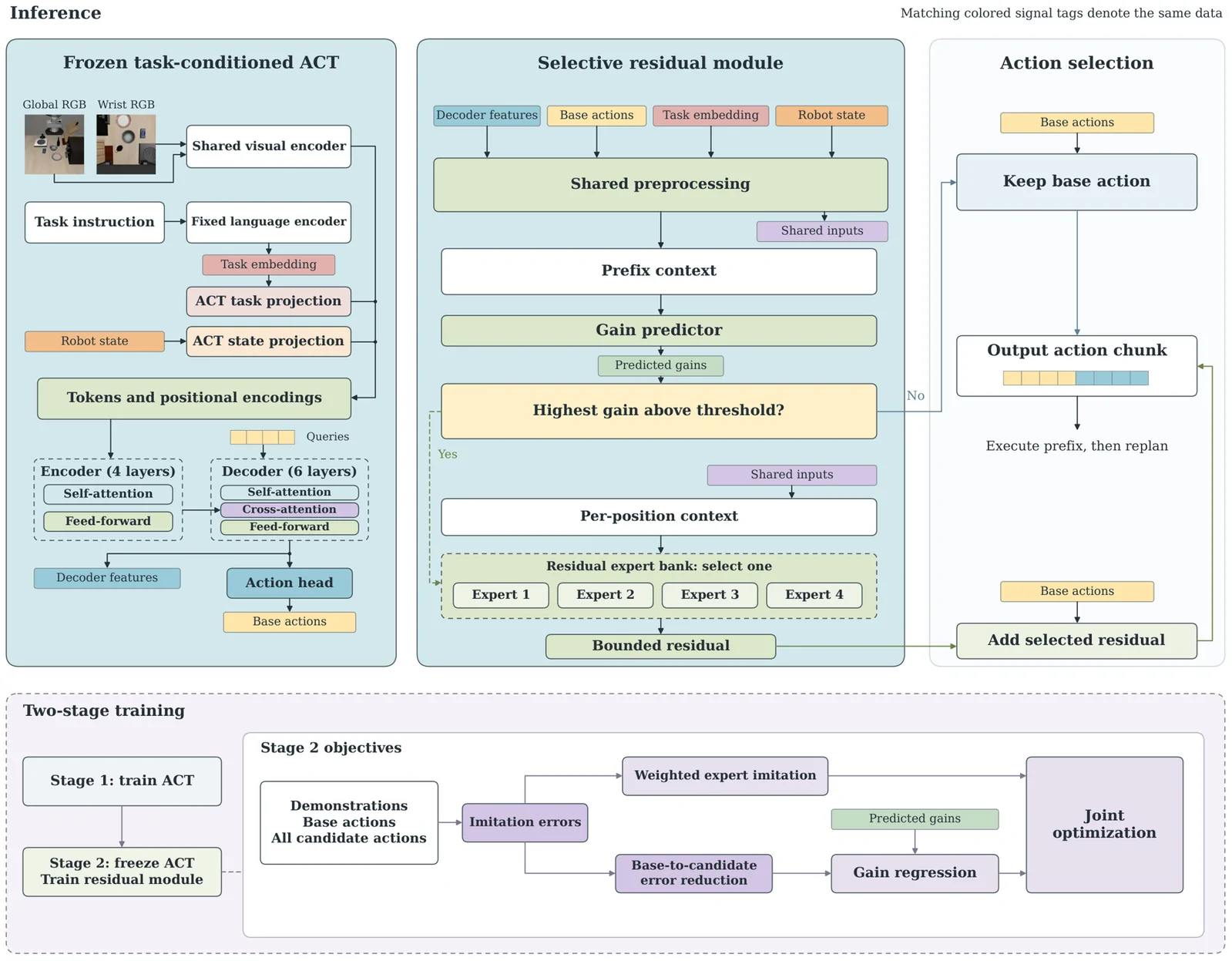
Overall framework of the selective residual action-chunking policy. The (**upper panel**) shows inference, and the (**lower panel**) shows the second-stage training objectives. Matching signal labels denote the same data. ACT: Action Chunking with Transformers.

As shown in Figure 1, the policy consists of a base policy, *K* bounded residual experts, and a gain predictor. The base policy produces an action chunk and decoder features. The experts construct corrections to this chunk, while the gain predictor uses the current task, state, decoder features, and base-action statistics to guide selection. Retaining the base action as an available output allows the policy to decide both which correction to apply and whether to apply one. The base policy is trained first and held fixed during the joint training of the residual experts and gain predictor.

### 3.2. Base Actions and Bounded Residual Candidates

The base policy is a task-conditioned ACT model, denoted by $\pi_{\theta }$. It uses an ImageNet-pretrained ResNet-18 visual backbone, decoder features of dimension $d_{f}=256$, a Transformer feed-forward dimension of 2048, and dropout of 0.1. A fixed all-MiniLM-L6-v2 encoder, based on Sentence-BERT [23] and MiniLM [24], produces a task representation of dimension $d_{e}=384$. After training, the base parameters are frozen and the model is kept in evaluation mode. Setting its action-sequence latent variable *z* to zero gives deterministic base actions $A_{t}^{0}$ and features from the final decoder layer $H_{t}\in R^{H\times d_{f}}$:

(2) $$\left(A_{t}^{0},H_{t}\right)=\pi_{\theta }\left(I_{t},q_{t},e_{m};z=0\right).$$

Equation (2) is used in both the second training stage and inference, giving the same base prediction for the same input. To condition corrections on the task, state, and prediction position, the expert input at position *j* is

(3) $$x_{t,j}=\mathrm{Concat}\left(\mathrm{LN}\left(H_{t,j}\right),\;P_{e}\left(e_{m}\right),\;P_{q}\left(q_{t}\right),\;a_{t,j}^{0}\right).$$

Here, $H_{t,j}$ and $a_{t,j}^{0}$ are the decoder feature and base action at position *j*, LN denotes layer normalization, and $P_{e}$ and $P_{q}$ are trainable projections of the task representation and proprioceptive state. These projections produce 64- and 32-dimensional vectors, respectively, so the expert input dimension is $d_{f}+64+32+d_{a}$. We use K=4 residual experts. Each is a multilayer perceptron with one hidden layer and one output layer with a hidden dimension of 256, an output dimension of $d_{a}$, and a GELU activation in the hidden layer. Expert parameters are independent across experts and shared across chunk positions within each expert. The residual and corrected action chunk for expert *k* are

(4) $$\Delta a_{t,j}^{k}=s⊙\tanh\left(E_{k}\left(x_{t,j}\right)\right),\;A_{t}^{k}=A_{t}^{0}+\Delta A_{t}^{k}.$$

The chunk $\Delta A_{t}^{k}$ stacks the position-wise residuals $\Delta a_{t,j}^{k}$. The vector $s\in R_{>0}^{d_{a}}$ specifies the residual scale for each action dimension, and ⊙ denotes element-wise multiplication. The hyperbolic tangent and scale vector bound the correction in normalized action space. Each expert’s output layer is initialized to zero, so the second training stage starts from the base prediction. The resulting *K* corrected candidates share the same base action and are subsequently selected using predicted gains.

### 3.3. Relative Gain Prediction and Selective Correction

We formulate candidate selection as gain prediction relative to the base action. Demonstration actions define the change in error for each candidate during training. During inference, the gain predictor estimates this change from the task and current state, determining whether to apply a residual or retain the base action.

#### 3.3.1. Gain Supervision from Imitation-Error Reduction

To balance the accuracy of the executed prefix with the quality of the complete prediction, we combine prefix and full-chunk imitation errors. Let $A_{t}^{*}$ be the demonstrated action chunk, and let $m_{j}\in \left\{0,1\right\}$ indicate valid positions, with zero assigned to padding. For a predicted chunk *A* and demonstration $A^{*}$, whose actions at position *j* are $a_{j}$ and $a_{j}^{*}$, the valid-prefix error over *r* positions is

(5) $$d_{r}\left(A,A^{*}\right)=\frac{\sum_{j=1}^{r}m_{j}{∥a_{j}-a_{j}^{*}∥}_{1}/d_{a}}{\max(1,\sum_{j=1}^{r}m_{j})}.$$

The combined error is

(6) $$d\left(A,A^{*}\right)=\lambda d_{h}\left(A,A^{*}\right)+\left(1-\lambda \right)d_{H}\left(A,A^{*}\right).$$

The coefficient λ∈[0,1] balances the two terms. The prefix term emphasizes the accuracy of the actions to be executed next, while the full-chunk term encourages accurate predictions over the entire action chunk. The target gain for expert *k* is

(7) $$g_{t,k}^{*}=\mathrm{sg}\;\left[d\left(A_{t}^{0},A_{t}^{*}\right)-d\left(A_{t}^{k},A_{t}^{*}\right)\right].$$

Here, sg denotes stop-gradient. A positive gain indicates that the candidate has lower imitation error than the base prediction on the current demonstration window; a negative gain indicates an increase in error. Retaining the base action corresponds to zero gain. All candidates are therefore evaluated on a common scale. Targets are recomputed as expert outputs change during training. Stop-gradient prevents the regression loss from propagating through its target values.

#### 3.3.2. Task- and State-Conditioned Gain Prediction

The gain predictor $G_{\psi }$ estimates expert gains from the current observation-conditioned representation, task information, and robot state. Visual information enters through the base decoder features, while proprioception is supplied explicitly. Its input combines decoder features over the execution prefix, base-action statistics, the task representation, and proprioception:

(8) $$c_{t}=\mathrm{Concat}\left({\bar{H}}_{t,1:h},\;P_{e}\left(e_{m}\right),\;P_{q}\left(q_{t}\right),\;{\bar{A}}_{t,1:h}^{0},\;\mathrm{Std}\left(A_{t,1:h}^{0}\right)\right).$$

The vector ${\bar{H}}_{t,1:h}$ is the mean of the layer-normalized decoder features over the first *h* positions. The quantities ${\bar{A}}_{t,1:h}^{0}$ and $\mathrm{Std}(A_{t,1:h}^{0})$ are the dimension-wise mean and population standard deviation of the base-action prefix. The predictor is a multilayer perceptron with one hidden layer and one output layer with input dimension $d_{f}+64+32+2d_{a}$, hidden dimension 128, and output dimension *K*. It uses a GELU hidden activation and a zero-initialized output layer. Its output ${\hat{g}}_{t}=G_{\psi }\left(c_{t}\right)$ contains one gain estimate per expert. Because this context does not require explicit residual candidates, gains can be predicted before the selected expert is evaluated.

#### 3.3.3. Chunk-Level Selection and Base-Action Retention

Action selection combines the maximum predicted gain with a threshold test. Let $k_{t}^{*}=\arg\max_{k}{\hat{g}}_{t,k}$. The output action chunk is

(9) $${\hat{A}}_{t}=\left\{\begin{array}{cc} A_{t}^{0}+\Delta A_{t}^{k_{t}^{*}}, & \max_{k}{\hat{g}}_{t,k}>\delta ,\\ A_{t}^{0}, & \mathrm{otherwise}. \end{array}\right.$$

The threshold δ≥0 is expressed on the same scale as the error gain in Equation (7). A positive threshold specifies a minimum predicted benefit for applying a correction. When the condition is satisfied, only the selected expert is evaluated, and that expert is used at all *H* positions. Otherwise, the policy bypasses the residual experts and outputs the base action. After executing the first *h* actions, the robot makes a new decision from updated observations. This rule maintains expert consistency within a chunk while permitting different branches at successive decision times.

### 3.4. Two-Stage Training and Joint Optimization

#### 3.4.1. Multi-Task Base-Policy Training

In the first stage, ACT is trained on the combined demonstrations of all tasks with an action-reconstruction loss and Kullback–Leibler (KL) regularization:

(10) $$L_{\mathrm{base}}=L_{\mathrm{rec}}+\beta_{\mathrm{KL}}L_{\mathrm{KL}}.$$

The reconstruction term $L_{\mathrm{rec}}$ is the action $L_{1}$ error with padded positions masked out. The KL term regularizes the variational distribution of the action-sequence latent, and $\beta_{\mathrm{KL}}$ sets its weight. Following model selection, the base policy is frozen. The second stage optimizes only the residual experts, gain predictor, and their shared projection and normalization layers.

#### 3.4.2. Soft Responsibilities for Residual Experts

To account for differences in candidate errors while retaining training coverage for all experts, we assign error-based soft responsibilities with a uniform component:

(11) $$w_{t,k}=\left(1-\epsilon \right)\frac{\exp[-\mathrm{sg}\left(d\left(A_{t}^{k},A_{t}^{*}\right)\right)/\tau ]}{\sum_{r=1}^{K}\exp\left[-\mathrm{sg}\left(d\left(A_{t}^{r},A_{t}^{*}\right)\right)/\tau \right]}+\frac{\epsilon }{K}.$$

The temperature τ>0 controls the concentration of the weights, and 0<ϵ<1 sets the uniform component. Experts with lower errors receive greater weights, while every expert retains a nonzero responsibility. These weights are computed from demonstration errors solely to allocate training supervision. Inference uses the selection rule in Equation (9).

#### 3.4.3. Gain Regression and the Joint Objective

The predictor is supervised by Equation (7) using a Smooth-$L_{1}$ loss with transition parameter *b*:

(12) $$L_{\mathrm{gain}}=E_{D}\left[\frac{1}{K}\sum_{k=1}^{K}{\mathrm{SmoothL}1}_{b}\left({\hat{g}}_{t,k},g_{t,k}^{*}\right)\right].$$

The second stage jointly minimizes the responsibility-weighted expert error and gain regression loss:

(13) $$L_{\mathrm{expert}}=E_{D}\left[\sum_{k=1}^{K}w_{t,k}d\left(A_{t}^{k},A_{t}^{*}\right)\right],\;L=L_{\mathrm{expert}}+L_{\mathrm{gain}}.$$

The expectations in Equations (12) and (13) are computed over action windows sampled from demonstrations. Expert networks receive gradients from $L_{\mathrm{expert}}$, the predictor receives gradients from $L_{\mathrm{gain}}$, and the shared projections and normalization receive gradients from both terms. The base policy remains unchanged. All candidates are evaluated during training to construct responsibilities and gain targets together; inference selects the output according to Equation (9). The two losses constrain candidate imitation accuracy and gain-estimation accuracy, respectively.

## 4. Experiments and Analysis

We evaluate the proposed method for multi-task manipulation with visual and proprioceptive feedback through simulation and real-world robot experiments. In simulation, we compare success rates and computational costs with MTACT and BAKU across all ten tasks in the LIBERO-Goal benchmark. Controls using the same frozen base, inference ablations, gain prediction evaluation, execution analyses, and hyperparameter sensitivity studies examine the roles of residual correction and selection and the factors affecting their performance. Real-world experiments use global and wrist RGB cameras with joint position feedback to further assess manipulation performance after joint training on three tasks.

### 4.1. Simulation Experiments

#### 4.1.1. Tasks and Evaluation Protocol

**Figure 2:**
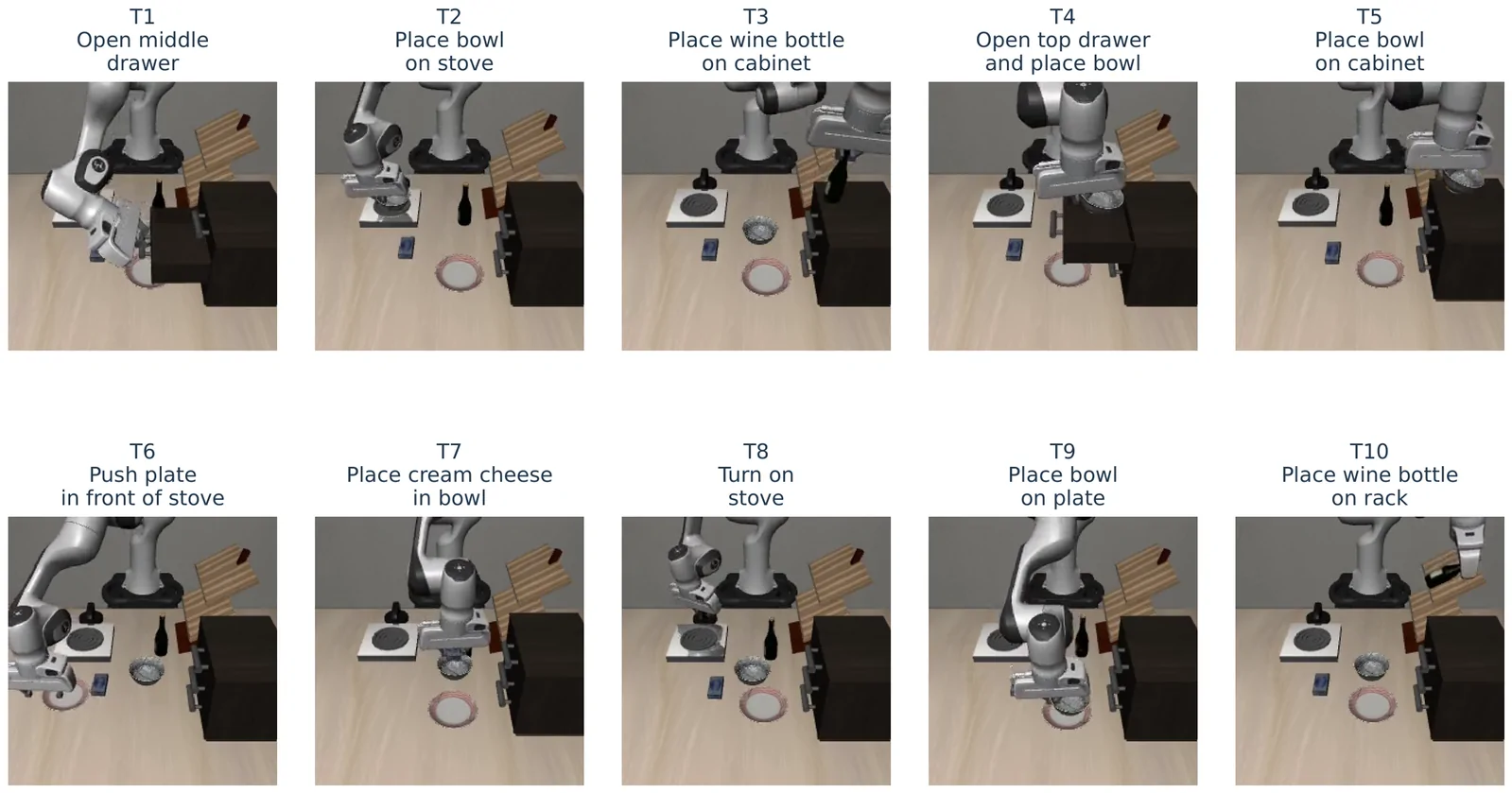
Manipulation examples from the ten LIBERO-Goal tasks.

Experiments cover all ten manipulation tasks in LIBERO-Goal [14]. We use the preprocessed demonstrations adopted in the OpenVLA experiments [25], with no-operation actions and trajectories that fail replay removed. The tasks share scene objects and initial-relation specifications but have different goal conditions; individual evaluations still vary in their specific object states. The dataset contains 428 trajectories, with 338 trajectories used for training and 90 for validation. Splits are made at the trajectory level within each task, and training covers all ten tasks. Figure 2 illustrates the manipulation objects and goals.

At each action-chunk prediction, the inputs comprise a global RGB view, a wrist RGB view, and the robot state. The proprioceptive state has dimension $d_{q}=8$, and the action contains end-effector translation, rotation, and gripper control, with dimension $d_{a}=7$. The action chunk length is H=75, and the first h=8 steps are executed at each update. Under this action representation, the residual experts and gain predictor have input dimensions of 359 and 366, respectively, and each residual expert outputs seven values per position.

The proposed method jointly trains one policy using demonstrations from all ten tasks, with task-language conditioning distinguishing the goals. Its total training budget is 1000 epochs: 500 for base-policy training, with the checkpoint selected using the validation set within this stage, followed by 500 for the residual experts and gain predictor with the selected base frozen. Both stages use AdamW with a batch size of eight, a learning rate of $10^{-4}$, and weight decay of $10^{-4}$. The visual backbone uses a learning rate of $5\times 10^{-5}$ in the first stage; the second stage clips the gradient norm at one. Training and feature computation run on an NVIDIA RTX 5090 GPU. After freezing the base, its decoder features and actions are precomputed for training the residual module. During deployment, they are computed from current observations.

In simulation, the residual scale is s=(1,1,1,1,1,1,2), the correction threshold is δ=0.01, and the execution prefix error weight is λ=0.75. The scale bounds corrections in normalized action space, with a larger allowance for the gripper dimension. The prefix and full-chunk error weights are 0.75 and 0.25, respectively, emphasizing the actions executed immediately while retaining a constraint on the full prediction. The positive threshold specifies a minimum predicted gain for applying a correction. The remaining training parameters are $\beta_{\mathrm{KL}}=10$, τ=0.05, ϵ=0.1, and b=0.05, specifying the base-policy KL weight, soft-responsibility temperature, uniform-mixture proportion, and gain regression Smooth-$L_{1}$ transition parameter, respectively.

The first-stage base and the MTACT baseline use the same network architecture, training configuration, and validation-based checkpoint-selection criterion. Our method selects and freezes a base checkpoint from the first 500 epochs. MTACT is trained for 1000 epochs, with its checkpoint selected by the same criterion over that training budget.

Each method is evaluated in 50 trials per task. We report success rates for individual tasks and their mean across the ten tasks. MTACT [10] and BAKU [11] are the comparison baselines. The three methods in Table 1 follow the same evaluation protocol and use the same dual-view inputs, task conditions, and demonstration split, with a training budget of 1000 epochs each.

#### 4.1.2. Multi-Task Manipulation Performance

As shown in Table 1, MTACT and BAKU achieve mean success rates of 79.8% and 87.0%, respectively. The proposed policy reaches 91.2%, the highest mean among the compared methods, improving on them by 11.4 and 4.2 percentage points. It completes 456 of 500 trials, corresponding to 57 more successes than MTACT and 21 more than BAKU.

The proposed policy attains the best or tied-best success rate on five of the ten tasks. It achieves 100% on placing the wine bottle on top of the cabinet, pushing the plate in front of the stove, and turning on the stove. For opening the top drawer and placing the bowl inside, its success rate is 98%, exceeding MTACT and BAKU by 20 and 36 percentage points. For placing the wine bottle on the rack, it achieves 86%, with respective improvements of 52 and 4 percentage points. Under the same sensing inputs and evaluation protocol, the proposed policy achieves the highest mean success rate among the compared methods. This supports selective action correction as an effective extension of the shared visuomotor policy.

We compare the training update and action-chunk inference times of the compared methods on the same RTX 5090 using FP32 precision (Table 2). Training updates use a batch size of eight, and inference uses a batch size of one.

The proposed policy requires 13.44 ms to predict an action chunk, compared with 11.88 ms for MTACT. Its additional inference computation comes from gain prediction and at most one residual expert. BAKU has the lowest measured inference time, while the proposed policy achieves the highest mean success rate among the compared methods.

### 4.2. Ablation Study

We first evaluate two controls to distinguish the first-stage policy from the effect of residual learning. The frozen base control generates actions using the task-conditioned ACT checkpoint selected on the validation set within the first 500 epochs, without residual correction. Single residual independently trains one residual network on top of this same frozen base and uses its output to correct the base actions. The full policy and the single-residual control share the frozen checkpoint. The latter does not use an expert bank or expert selection and is trained independently, rather than extracted from the jointly trained experts.

We additionally define three variants to examine inference-time expert selection and base-action fallback.

1. No fallback retains predicted gain-based expert selection but removes the base-action branch. The selected expert’s correction is always applied, regardless of whether its predicted gain exceeds the threshold.
2. Fixed expert selects the expert with the lowest mean action error on the validation set and always uses it for residual correction across all tasks, with base-action fallback disabled.
3. Random routing uniformly samples one of the four experts at each chunk prediction and applies its residual without base-action fallback.

All three variants use the full policy’s trained weights and change only the inference rule.

#### 4.2.1. Overall Ablation Results

Table 3 shows that the frozen first-stage base achieves a mean success rate of 70.0%, whereas the independently trained single-residual policy reaches 87.0%, an increase of 17.0 percentage points. This comparison demonstrates the benefit of learning action corrections on top of the frozen policy. The full policy achieves 91.2%, exceeding the frozen base and the single-residual control by 21.2 and 4.2 percentage points, respectively. The latter improvement reflects the combined effect of multiple-expert learning and selective correction.

Removing fallback, fixing the expert, and using random routing yield mean success rates of 89.4%, 89.0%, and 86.6%, respectively: decreases of 1.8, 2.2, and 4.6 percentage points from the full policy. Combining gain-guided expert selection with base-action fallback therefore provides the best overall performance among these variants. With gain-guided selection retained, adding base-action fallback increases the mean from 89.4% to 91.2%, demonstrating the benefit of retaining the base action. When fallback is disabled in both configurations, predicted gain selection exceeds random routing by 2.8 percentage points. Thus, selecting experts according to the task and state also improves mean success. Combining the two mechanisms gives the highest mean success rate among the compared configurations.

Furthermore, the full policy’s advantages over No fallback and Fixed expert are concentrated primarily in T10. This task requires transitioning a slender wine bottle from an upright grasp to a placement configuration suited to an inclined support, making coordination among position, orientation, and contact particularly important. Rotating a slender object changes both its orientation and the spatial positions of its ends, so orientation adjustment must be coordinated with translation. In this task, the policy progresses through approach, grasping and lifting, orientation adjustment, and approach to the rack followed by placement. Approach and grasping emphasize gripper-to-bottle alignment; transport involves coordinated position and orientation adjustments; placement requires coordinating the bottle’s position relative to the inclined rack and the ensuing contact. These stage-specific requirements illustrate how control demands change substantially over the course of T10. The relative suitability of base actions and residual corrections may therefore vary with state: some action chunks can retain the base prediction, whereas others require further adjustment. Selective correction enables this alternation, consistent with the larger improvement observed in T10.

Benefits on other tasks are constrained by existing performance and the quality of candidate corrections. In T1, T2, T3, T4, T6, T8, and T9, No fallback and Fixed expert already achieve success rates of 94% to 100%, leaving limited room for improvement. In T7, the full policy and these two variants achieve 64% to 66%. This task primarily involves grasping the cream cheese, transporting it, and placing it in the bowl, with control requirements centered on grasping and alignment with the target location. Compared with reorienting a slender object and placing it on an inclined support in T10, these requirements may vary less over the course of execution, potentially leaving less scope for additional gains from selective correction.

#### 4.2.2. Gain Prediction and Correction Effectiveness

We directly evaluate the gain predictor on validation demonstrations by comparing predicted gains with gains computed from action imitation errors and measuring the error of the full policy’s output. The error metric follows Equation (6), and the actual gain is the difference between the base-action error and the expert-corrected action error. Gain mean absolute error (MAE) measures numerical prediction error. Gain-sign balanced accuracy averages recall for positive and nonpositive gains, using zero as the classification threshold. Pairwise ranking accuracy measures the proportion of expert-pair comparisons in which the ordering of predicted gains agrees with that of actual gains, evaluated among the four residual experts. Full-policy action error is evaluated using the expert selection and base-action-retention rule in Equation (9).

As shown in Table 4, gain prediction MAE is 0.03167, below the zero-gain reference of 0.03591. Gain-sign balanced accuracy reaches 64.98%, exceeding the uninformative reference of 50.00%, indicating that the predictor uses current observations and task conditions to distinguish the direction of candidate gains. Pairwise ranking accuracy is 65.83%, exceeding the random ordering reference of 50.00% by 15.83 percentage points. Predicted gains therefore provide useful information for comparing the relative quality of candidate corrections. Under the full policy’s selection rule, mean imitation error decreases from 0.33896 for the base actions to 0.31603, a relative reduction of 6.76%. Thus, selective correction reduces average action error on the validation demonstrations.

Imitation error and task success measure local action-prediction quality and goal completion over an entire trajectory, respectively. The former measures deviation from reference actions at demonstration states; the latter also depends on changing states, contact interactions, and accumulated errors during closed-loop execution. The reduction in imitation error in Table 4 and the increase in success rate in Table 3 both favor the full policy over the frozen base. Retaining the base-action branch also increases closed-loop success from 89.4% to 91.2%, supporting the practical benefit of applying corrections selectively. These findings support the method at both the action-prediction and closed-loop levels. Gain serves as an imitation-learning criterion for selecting corrections, whose contribution to task completion is evaluated through closed-loop trials.

#### 4.2.3. Branch Usage Across Tasks

**Figure 3:**
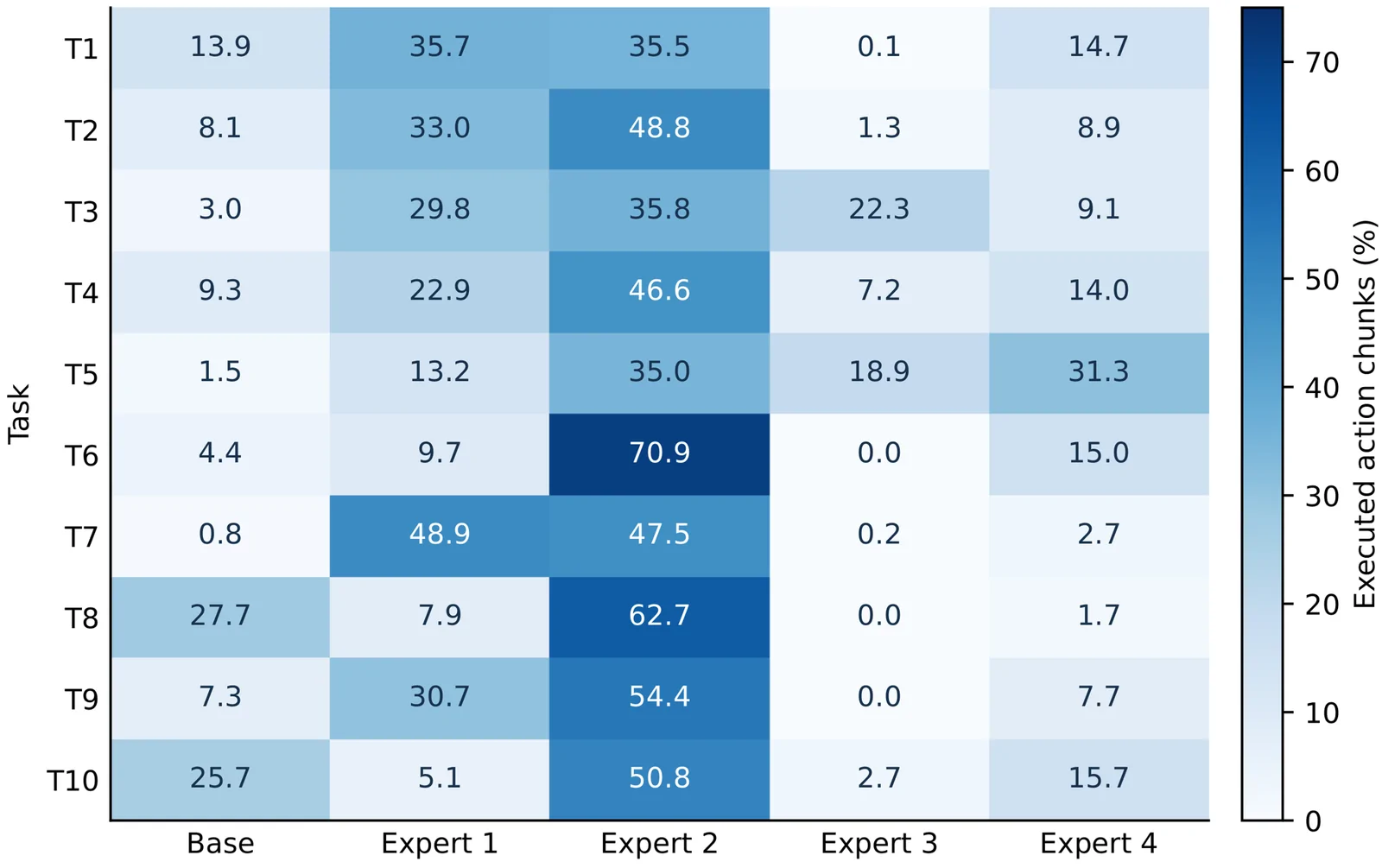
Action-chunk selection proportions (%) by task. Base denotes retaining the base action; Experts 1–4 denote residual branches. Each row is normalized within its task.

Figure 3 presents the distribution of chunk-level branch selections during execution, normalized by the number of action chunks within each task.

Across 7975 decisions, the base action is retained 792 times, or 9.9%. The four experts account for 24.3%, 48.0%, 5.0%, and 12.7%, respectively. Expert usage is uneven, with Expert 2 selected in nearly half of all decisions. Preferences also vary by task: plate pushing mainly uses Expert 2, whereas placing cream cheese in the bowl primarily uses Experts 1 and 2. The router therefore exhibits task-dependent expert selection preferences.

Base-action retention also varies across tasks. Its proportion is 27.7% for turning on the stove and 25.7% for placing the wine bottle on the rack, compared with 1.5% for placing the bowl on top of the cabinet. The latter task therefore uses corrected candidates for most decisions. On the wine-bottle-on-rack task, retaining the fallback branch increases success from 66% to 86%. Together, branch-use statistics and ablations illustrate how base-action retention and residual correction can complement one another in multi-task action selection.

#### 4.2.4. Expert Selection Preferences

**Figure 4:**
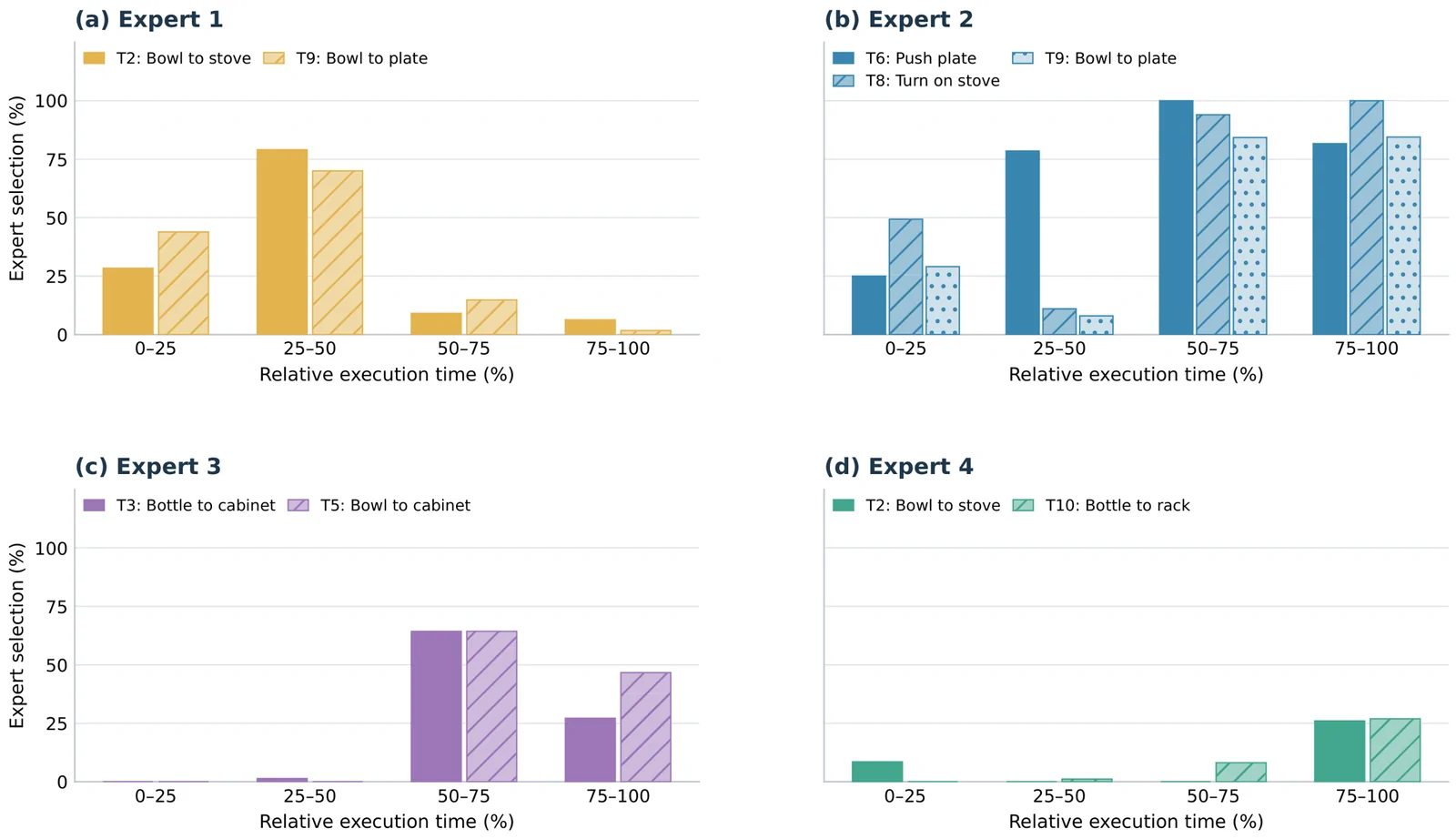
Expert selection proportions across execution intervals in successful trajectories of representative tasks. Task IDs follow Table 1.

**Figure 5:**
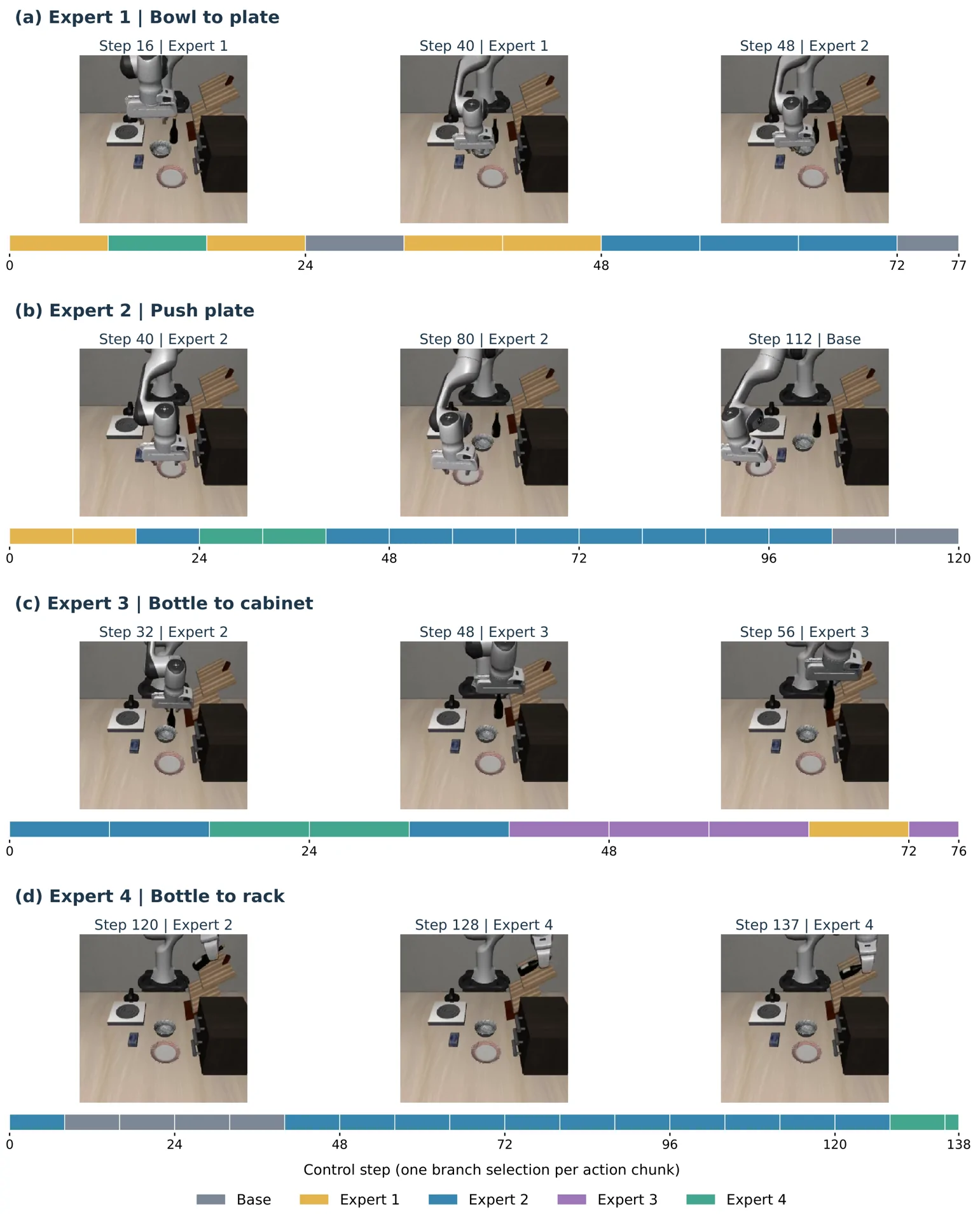
Representative trajectories illustrating the selection of Experts 1–4, with branch selection timelines. Rows follow the expert order in Figure 4; color bars indicate the branches used during execution.

To examine how expert selection relates to the manipulation process, we analyze all successful trajectories of representative tasks. Each action chunk is assigned to one of four relative-time intervals according to its start time divided by the trajectory duration. Within each task and interval, we compute the fraction of all branch selections assigned to the expert of interest. Figure 4 presents the statistics in the order of Experts 1–4, and Figure 5 provides representative trajectories in the same order. By comparing expert selection proportions across time intervals and examining the manipulation processes in representative trajectories, we analyze how expert selection changes as tasks progress.

Expert 1 exhibits a similar early-to-middle execution preference in two bowl-manipulation tasks. In the 25–50% interval of bowl-to-stove and bowl-to-plate placement, its selection proportions are 79.1% and 70.0%, respectively, and decrease thereafter (Figure 4a). In the example in Figure 5a, Expert 1 is selected as the end effector approaches the bowl and adjusts toward a grasping position, followed by a switch to Expert 2. Despite their different placement goals, the two tasks exhibit a shared selection preference during comparable approach and grasp-preparation segments.

Expert 2 has high selection proportions in the second half of several tasks. It accounts for 100% of selections in the 50–75% interval of plate pushing, and 100% and 84.5% in the final quarter of turning on the stove and bowl-to-plate placement, respectively (Figure 4b). Expert 2 participates in continuous pushing, manipulation near the stove knob, and transport toward a target after grasping. Figure 5b illustrates sustained Expert 2 use during plate pushing, followed by base-action retention near the end. Its use thus spans different objects and goals and varies with the execution process.

Expert 3 exhibits a more concentrated transport-related preference in the two cabinet-top placement tasks. Its selection proportions are low in the first half of bottle-to-cabinet and bowl-to-cabinet placement, increasing to 64.3% and 64.4% in the 50–75% interval (Figure 4c). In the bottle-transport example in Figure 5c, the policy repeatedly selects Expert 3 as the object is lifted and moved toward the cabinet top. Similar temporal distributions and transport motions involving different objects indicate a shared selection preference across tasks.

Expert 4 shows more task-dependent preferences. In bowl-to-stove and bottle-to-rack placement, its proportions in the final quarter are 26.0% and 26.9%, higher than in the other intervals of each task (Figure 4d). Figure 5d shows a switch from Expert 2 to Expert 4 as the bottle approaches the target position on the rack. Expert 4 also appears during early approach in bottle-to-cabinet placement, indicating that the same expert can participate in different execution segments across tasks.

**Figure 6:**
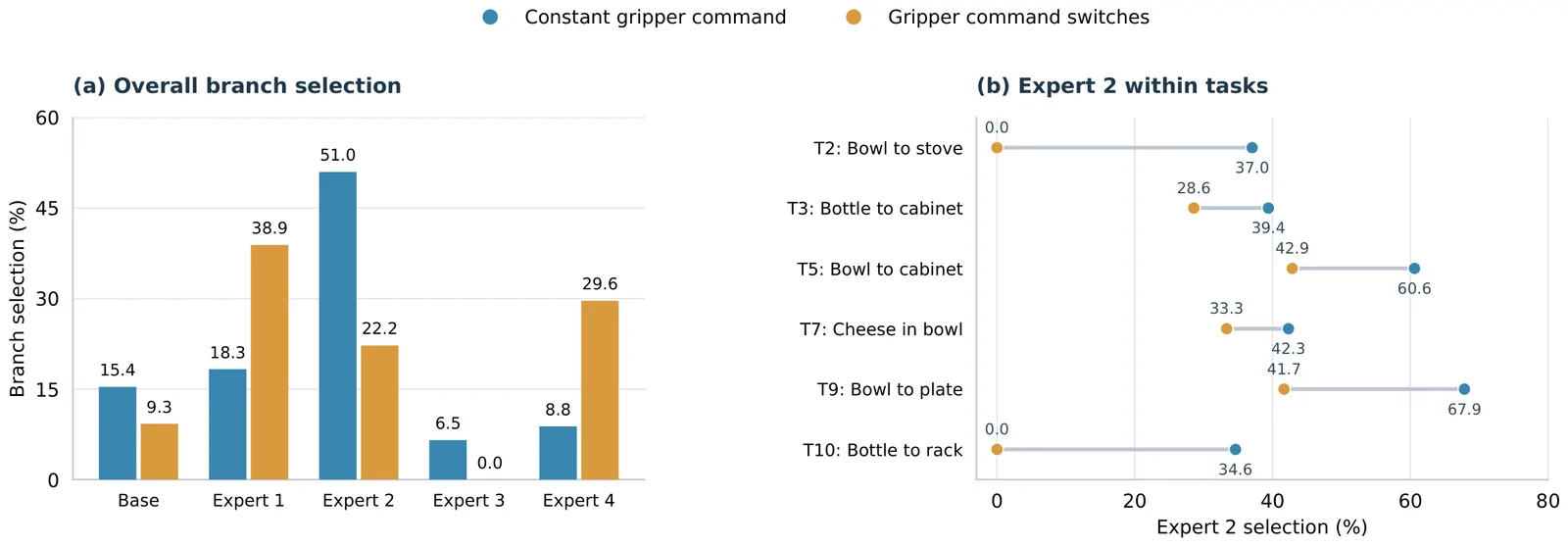
Branch selection under different demonstrated gripper-command conditions. (**a**) Overall branch selection proportions. (**b**) Expert 2 selection within tasks; connected points represent the two groups for the same task.

We further examine the association between expert selection and gripper commands in 360 offline demonstration validation windows. Based on whether the gripper command switches within the first eight demonstrated actions, we divide the windows into constant-command (306) and switching-command (54) groups. Figure 6a compares branch selection proportions across these groups. Figure 6b compares Expert 2 selection within the six tasks having at least five windows in each group.

Expert 2 is selected in 51.0% of constant-command windows and 22.2% of switching-command windows (Figure 6a). Its selection rate is also higher in the constant-command group in all six tasks shown in Figure 6b, indicating that the observed preference occurs across multiple tasks. Experts 1 and 4 instead have higher overall selection rates in the switching-command group, at 38.9% and 29.6%, compared with 18.3% and 8.8% in the constant-command group. Expert 4’s switching association mainly arises from bowl-to-stove and bottle-to-rack placement. This auxiliary analysis complements the trajectory observations from the perspective of demonstrated actions, linking expert selection to gripper-command changes within action segments.

Together, the evidence indicates that expert selection exhibits preferences associated with the task and manipulation process: similar segments can share preferences, while the same expert can participate in different stages across tasks. These preferences reflect how conditional residual selection varies with execution context.

#### 4.2.5. Hyperparameter Sensitivity

We use one-factor-at-a-time experiments to examine residual magnitude, correction threshold, and execution prefix weighting. Thresholds are 0, 0.01, and 0.02; the full policy’s trained weights are fixed while only the inference threshold changes. Residual scales are multiplied by 0.5, 1.0, or 1.5 relative to the default vector, and prefix-error weights are 0.5, 0.75, or 1.0. The scale and prefix weight variants share the same frozen base and are each trained for 500 second-stage epochs, with other settings unchanged. Each configuration is evaluated in 50 trials per task.

**Figure 7:**
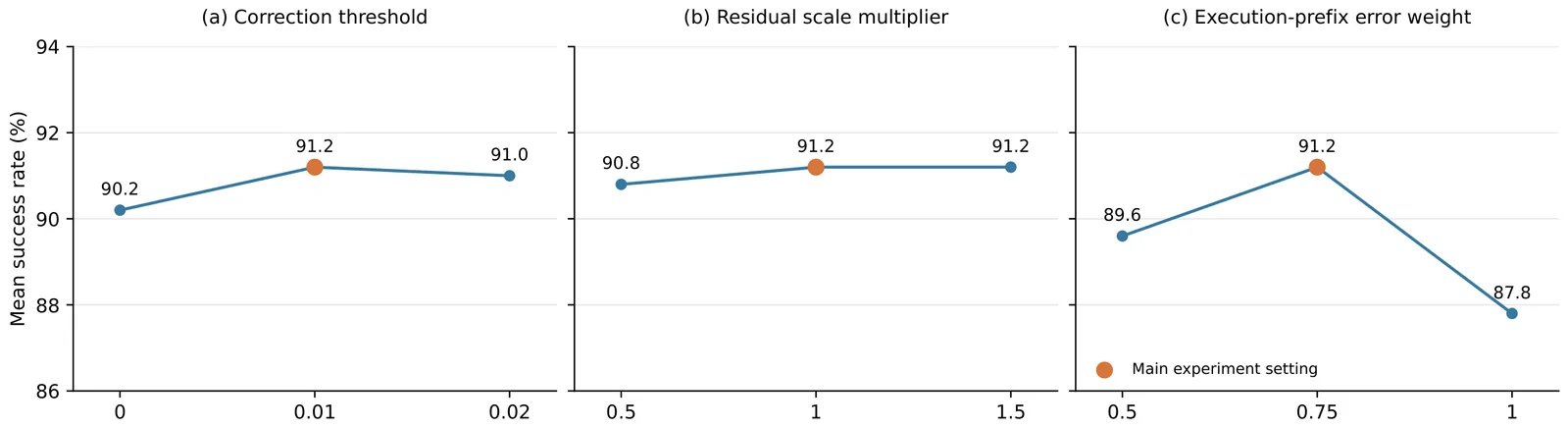
Sensitivity to the correction threshold, residual scale multiplier, and prefix-error weight. Each panel shows the mean success rate. Orange markers indicate the settings used in the main experiments of this study.

As shown in Table 5 and Figure 7, mean success rates range from 90.2% to 91.2% across the tested correction thresholds and from 90.8% to 91.2% across the tested residual-scale multipliers, indicating relatively small changes within these ranges.

To further examine how prefix weighting affects expert selection, Table 6 reports the branch selection proportions of each configuration during closed-loop execution.

Increasing the prefix weight from 0.5 to 0.75 changes the overall frequency of residual correction only slightly, but substantially changes the distribution of expert use. Base-action retention decreases from 11.2% to 9.9%, while Expert 2 accounts for approximately half of all decisions in both configurations. Meanwhile, Expert 1 selection increases from 6.9% to 24.3%, and Expert 3 selection decreases from 17.8% to 5.0%. Thus, the two configurations apply residual corrections at similar overall frequencies but exhibit different preferences among candidate experts.

When the prefix weight increases to 1.0, base-action retention rises to 31.2%, indicating less frequent use of residual correction. Among decisions that apply a residual, Expert 1 accounts for approximately 70.5%, compared with 53.3% for Expert 2, the most frequently selected expert at a weight of 0.75. The configuration trained with prefix error alone therefore applies corrections less often and relies more heavily on a single expert when it does. A prefix weight of 0.75 achieves 91.2% mean success, compared with 89.6% and 87.8% at weights of 0.5 and 1.0, respectively. Together, these results indicate that emphasizing the execution prefix while retaining full-chunk supervision benefits manipulation performance under the evaluated settings.

### 4.3. Real-World Experiments

#### 4.3.1. Sensing and Actuation Setup and Tasks

We use a Hiwonder NexArm tabletop manipulator to evaluate multi-task execution with real visual observations and physical contact. The arm has five motion joints and an independently actuated gripper, with joint position feedback and position-command control. The real-world policies predict joint-space actions. Both state and action have six dimensions, corresponding to the five joint positions and gripper position.

**Figure 8:**
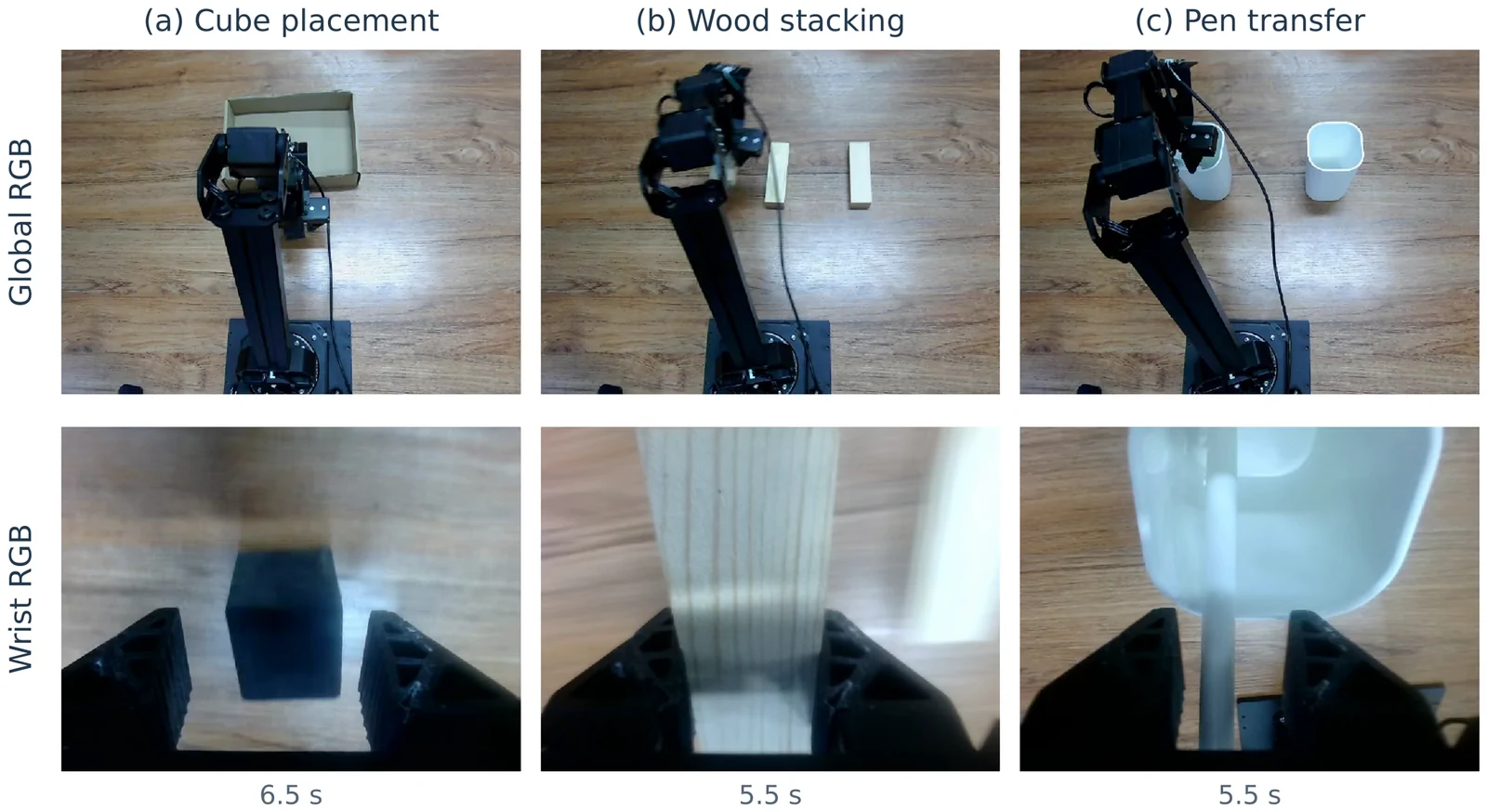
Dual-view observations for three NexArm tasks. The upper row shows the global RGB view and the lower row the wrist RGB view.

A global RGB camera and a wrist-mounted RGB camera provide complementary views of the workspace and the region near the gripper. Figure 8 shows example observations for the three tasks. Demonstration images have a resolution of 640×480, and videos are recorded at 30 frames per second. The global view captures the arrangement of objects and destinations, and the wrist view captures the local scene as the gripper moves. Joint position feedback supplies the current robot configuration. At each action-chunk prediction, these observations and the task instruction condition an action chunk; its prefix is sent as position commands before the next prediction is formed from updated observations. The policy therefore connects camera observations and proprioceptive feedback to arm and gripper actuation throughout task execution.

**Figure 9:**
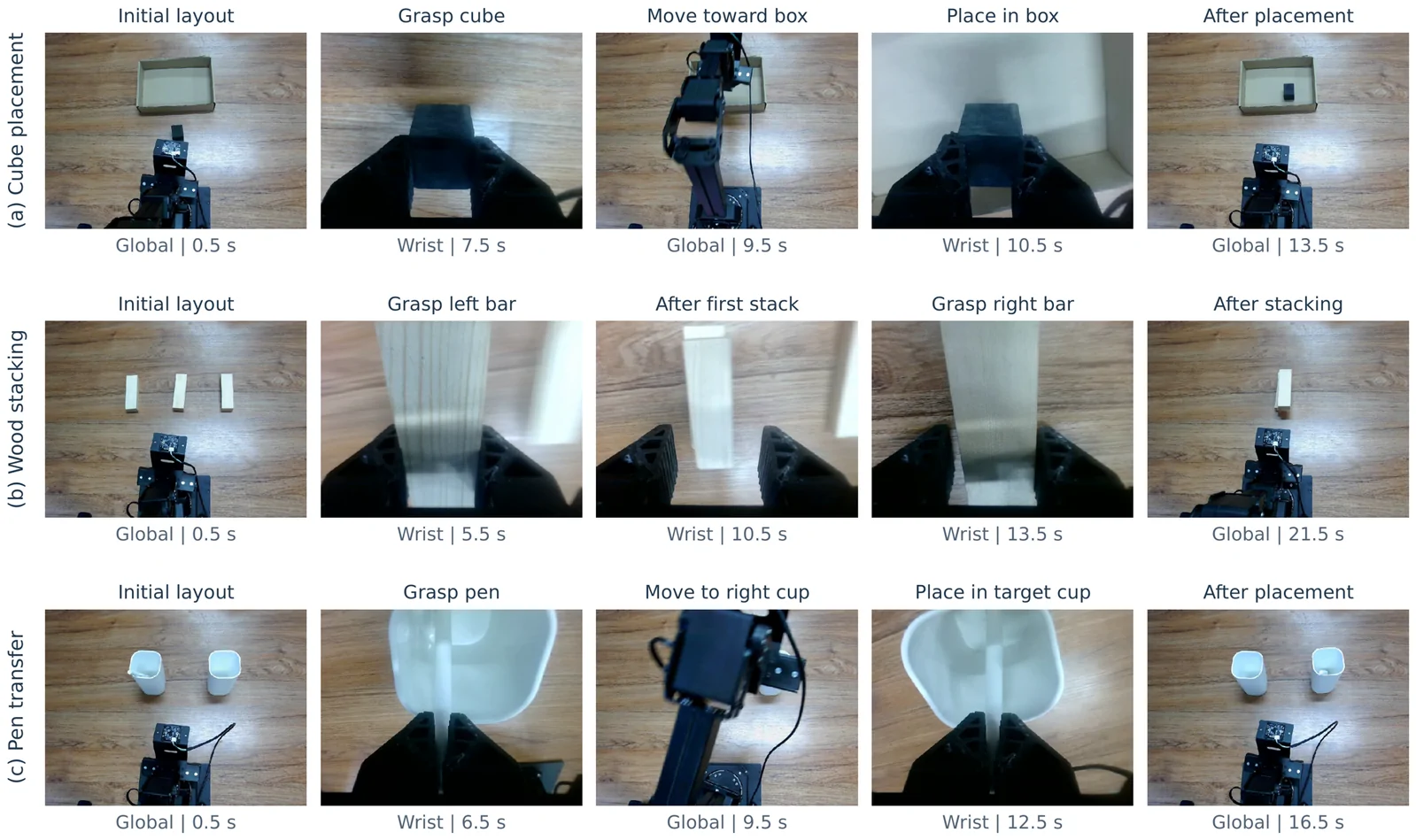
Training demonstrations for the three NexArm tasks. Each row presents key frames in temporal order.

The tasks are cube placement, wooden bar stacking, and pen transfer, illustrated in Figure 9. Cube placement requires grasping a cube, moving it over a box, and releasing it inside. Wooden bar stacking requires placing the wooden bars from both sides onto the central bar in sequence, accounting for the existing stack when placing the second bar. Pen transfer requires removing a pen from the left cup and placing it inside the right cup. The three tasks share the same robot and dual-view observation setup. Together, they assess localization, grasping, and placement in three settings: placement into a container, sequential manipulation of multiple objects, and transfer of a slender object.

We collect 50 demonstration trajectories per task and split them into training and validation sets at an 80:20 ratio. This gives 40 training and 10 validation trajectories per task, totaling 120 training and 30 validation trajectories across the three tasks. MTACT and the proposed method each learn one unified policy covering all three tasks, with task instructions distinguishing their goals. Each method is evaluated in 50 trials per task, for 300 real-world trials in total. We report closed-loop task success rates and the equally weighted mean across the three tasks.

#### 4.3.2. Multi-Task Manipulation Performance

As shown in Table 7, after joint training on the three real-world tasks, the proposed policy achieves a mean success rate of 78.7%, exceeding MTACT’s 68.7% by 10.0 percentage points. It performs better on all three tasks. With the same global and wrist camera inputs and proprioceptive feedback, selective residual correction improves the overall performance of the shared policy. The results support its use for real-world vision-guided multi-task manipulation.

## 5. Discussion and Limitations

The improvements over MTACT in simulation and physical robot operation support selective residual learning for multi-task visuomotor control. The base ACT generates actions from dual-view RGB observations, proprioceptive state, and task information; residual experts refine these predictions for the current execution context. Freezing the base model concentrates second-stage learning on action adjustment while preserving the learned observation-to-action mapping.

Comparisons using the same frozen base show that an independently trained residual network improves the base policy’s performance, while the full method further increases the success rate. This improvement reflects both learning multiple residual experts and applying their corrections selectively.

With the trained weights held fixed, disabling base-action fallback or using fixed or random expert selection reduces mean success in the ablation study. This supports incorporating both expert selection and base-action retention into residual correction. Predicted gains are learned relative to the imitation error of the base actions, providing a common basis for comparing candidate corrections and deciding whether to retain the base prediction. Expert selection analyses further reveal branch-use preferences associated with different tasks and manipulation processes, consistent with the design of adapting correction choices to current observations and task information.

On validation demonstrations, the gain predictor’s gain-sign balanced accuracy and pairwise ranking accuracy both exceed their respective reference levels, and the full policy reduces mean imitation error. Gain estimation and residual expressiveness remain limiting factors. Offline imitation error differs from closed-loop outcomes, which also depend on object interactions, accumulated errors, and encountered states. Lower imitation error need not increase task success, and performance remains below the MTACT baseline on some tasks. Bounded residuals limit the magnitude of action adjustments, and their ability to compensate for errors depends on the quality of the base predictions. The modest mean improvement over fixed-expert selection also suggests that candidate complementarity and selection quality remain open directions for refinement.

The hyperparameters have not yet been systematically optimized. The current sensitivity analysis covers only a limited set of parameter values, leaving their optimal combination and interactions for further investigation. Future work could conduct more systematic joint hyperparameter search to identify combinations that better balance correction capacity and closed-loop performance.

## 6. Conclusions

This work presents a selective residual action-chunking policy for vision-guided multi-task robotic manipulation, conditioned on global and wrist RGB observations, proprioceptive state, and task information. After training, the parameters of the task-conditioned ACT remain frozen, while bounded residual experts and a gain predictor learn to refine its predictions. Gain-guided selection determines whether to apply a residual correction or retain the base action, and receding-horizon execution incorporates new observations into subsequent action predictions.

Across all ten tasks in the LIBERO-Goal benchmark, the method achieves a mean success rate of 91.2%, exceeding MTACT and BAKU by 11.4 and 4.2 percentage points, respectively. When jointly trained on three real-world tasks, it achieves 78.7%, exceeding MTACT by 10.0 percentage points. Additional controlled experiments and analyses evaluate the proposed method in terms of component contributions, gain prediction, and execution behavior, among other aspects. These results show that selectively correcting action predictions based on visual observations, proprioceptive state, and task information can improve the overall performance of ACT in multi-task robotic manipulation. 

## Figures and Tables

**Table 1:** Per-task success rates (%) on LIBERO-Goal. Each method is evaluated in 50 trials per task. MTACT: multi-task Action Chunking with Transformers.

Task	BAKU	MTACT	Proposed
T1 Open the middle drawer	74.0	100.0	92.0
T2 Place the bowl on the stove	96.0	98.0	94.0
T3 Place the wine bottle on top of the cabinet	92.0	92.0	100.0
T4 Open the top drawer and place the bowl inside	62.0	78.0	98.0
T5 Place the bowl on top of the cabinet	98.0	90.0	82.0
T6 Push the plate in front of the stove	92.0	76.0	100.0
T7 Place the cream cheese in the bowl	76.0	36.0	64.0
T8 Turn on the stove	100.0	100.0	100.0
T9 Place the bowl on the plate	98.0	94.0	96.0
T10 Place the wine bottle on the rack	82.0	34.0	86.0
Mean success rate	87.0	79.8	91.2
Successful trials/total trials	435/500	399/500	456/500

**Table 2:** Training update and action-chunk inference times. For the proposed policy, training update times are listed as stage one/stage two; inference time refers to the final policy. Stage two updates only the residual module using precomputed base features and excludes feature precomputation.

Method	Training Update (ms)	Action-Chunk Inference (ms)
BAKU	21.48	4.41
MTACT	45.61	11.88
Proposed	45.61/3.44	13.44

**Table 3:** Per-task success rates (%) for the frozen base, the independently trained single-residual control, and inference-time ablations. Each configuration is evaluated in 50 trials per task. Task IDs correspond to Table 1.

Task	Frozen Base	Single Residual	Full Policy	No Fallback	Fixed Expert	Random Routing
T1	92.0	84.0	92.0	94.0	98.0	80.0
T2	92.0	92.0	94.0	94.0	94.0	92.0
T3	98.0	100.0	100.0	100.0	98.0	100.0
T4	66.0	92.0	98.0	98.0	94.0	94.0
T5	40.0	82.0	82.0	80.0	80.0	76.0
T6	78.0	90.0	100.0	100.0	100.0	98.0
T7	26.0	64.0	64.0	64.0	66.0	64.0
T8	100.0	100.0	100.0	100.0	100.0	100.0
T9	90.0	98.0	96.0	98.0	98.0	94.0
T10	18.0	68.0	86.0	66.0	62.0	68.0
Mean	70.0	87.0	91.2	89.4	89.0	86.6
Successes	350/500	435/500	456/500	447/500	445/500	433/500

**Table 4:** Gain prediction and action correction on validation demonstrations.

Metric	Result	Reference
Gain prediction MAE	0.03167	Zero-gain prediction: 0.03591
Gain-sign balanced accuracy	64.98%	Uninformative reference: 50.00%
Pairwise ranking accuracy	65.83%	Random ordering reference: 50.00%
Mean full-policy imitation error	0.31603	Base action: 0.33896

**Table 5:** Hyperparameter sensitivity analysis on LIBERO-Goal.

Parameter	Value	Success (%)
Threshold	0	90.2
Threshold	0.01	91.2
Threshold	0.02	91.0
Scale multiplier	0.5	90.8
Scale multiplier	1	91.2
Scale multiplier	1.5	91.2
Prefix weight	0.5	89.6
Prefix weight	0.75	91.2
Prefix weight	1	87.8

**Table 6:** Branch selection proportions and success rates (%) under different prefix weights.

Prefix Weight	Base Retained	Expert 1	Expert 2	Expert 3	Expert 4	Success
0.50	11.2	6.9	49.2	17.8	14.8	89.6
0.75	9.9	24.3	48.0	5.0	12.7	91.2
1.00	31.2	48.5	8.4	5.2	6.7	87.8

**Table 7:** Real-world multi-task success rates (%) on NexArm. Each task has 40 training and 10 validation demonstrations, and each method is evaluated in 50 trials per task.

Task	MTACT	Proposed
Pick up the cube and place it in the box	76.0	84.0
Stack the two side bars onto the central wooden bar	66.0	78.0
Transfer the pen from the left cup into the right cup	64.0	74.0
Mean success rate	68.7	78.7
Successful trials/total trials	103/150	118/150

## Data Availability

The original contributions presented in this study are included in the article. Further inquiries can be directed to the corresponding author.
